# mGem: Progress on the development of conjugate vaccines for *Cryptococcus neoformans* infections

**DOI:** 10.1128/mbio.03538-25

**Published:** 2026-03-10

**Authors:** Piotr R. Stempinski, Samuel Rodrigues Dos Santos Junior, Arturo Casadevall

**Affiliations:** 1 W. Harry Feinstone Department of Molecular Microbiology and Immunology, The Johns Hopkins Bloomberg School of Public Health 25802, Baltimore, Maryland, USA; Vallabhbhai Patel Chest Institute, Delhi, India

**Keywords:** *Cryptococcus neoformans*, conjugate vaccines, cryptococcosis, glucuronoxylomannan (GXM), vaccine design, polysaccharide-protein conjugates, fungal vaccines

## Abstract

*Cryptococcus neoformans* is an important fungal pathogen that causes cryptococcosis, a life-threatening disease predominantly affecting immunocompromised individuals. Current estimates suggest that cryptococcosis is responsible for over 150,000 deaths annually, highlighting the need for the development of effective preventive measures. Early research utilizing polysaccharide filtrates (CneF) laid the groundwork for polysaccharide-containing vaccines, demonstrating partial protection in murine infection models. Further vaccine constructs made with purified extracellular polysaccharides (EPS) conjugated to protein carriers elicited strong antibody responses and enhanced survival. Recently, more attention was given to the purified capsule components glucuronoxylomannan (GXM) and glucuronoxylomannogalactan (GXMGal), with studies indicating that GXM conjugates can elicit protective effects. The development of semisynthetic polysaccharide-based vaccines offers a new approach to addressing lot-to-lot variability, enhancing consistency. In conclusion, strategies that advance from early crude formulations to more sophisticated conjugate and synthetic vaccines hold great promise for developing an effective vaccine to prevent and treat cryptococcal diseases.

## PERSPECTIVE

*Cryptococcus neoformans* and *Cryptococcus gattii* are fungal pathogens that cause cryptococcosis, a life-threatening disease manifesting as meningitis (1–4). Cryptococcosis affects mainly immunocompromised individuals with advanced HIV/AIDS, solid organ transplant recipients, and patients undergoing immunosuppressive therapies (1). Current estimates suggest that cryptococcal meningitis is responsible for over 150,000 AIDS-related deaths annually, mostly in Sub-Saharan Africa (3). To highlight *Cryptococcus* spp. as an urgent threat, the World Health Organization has indicated *C. neoformans* as a critical-priority fungal pathogen (5, 6). With limited treatment options, the development of a preventive vaccine is considered the most promising strategy to reduce disease burden and transmission (7, 8). However, despite decades of research, no licensed vaccine is available against *Cryptococcus* spp. or any other fungal pathogen. The complexity of the fungal cell, together with the fact that cryptococcosis usually occurs in immunocompromised individuals, has hindered progress (9). Nonetheless, various approaches and advances in conjugate vaccine technology, especially those targeting the cryptococcal polysaccharide capsule, show potential for the development of a functional vaccine.

**Fig 1:**
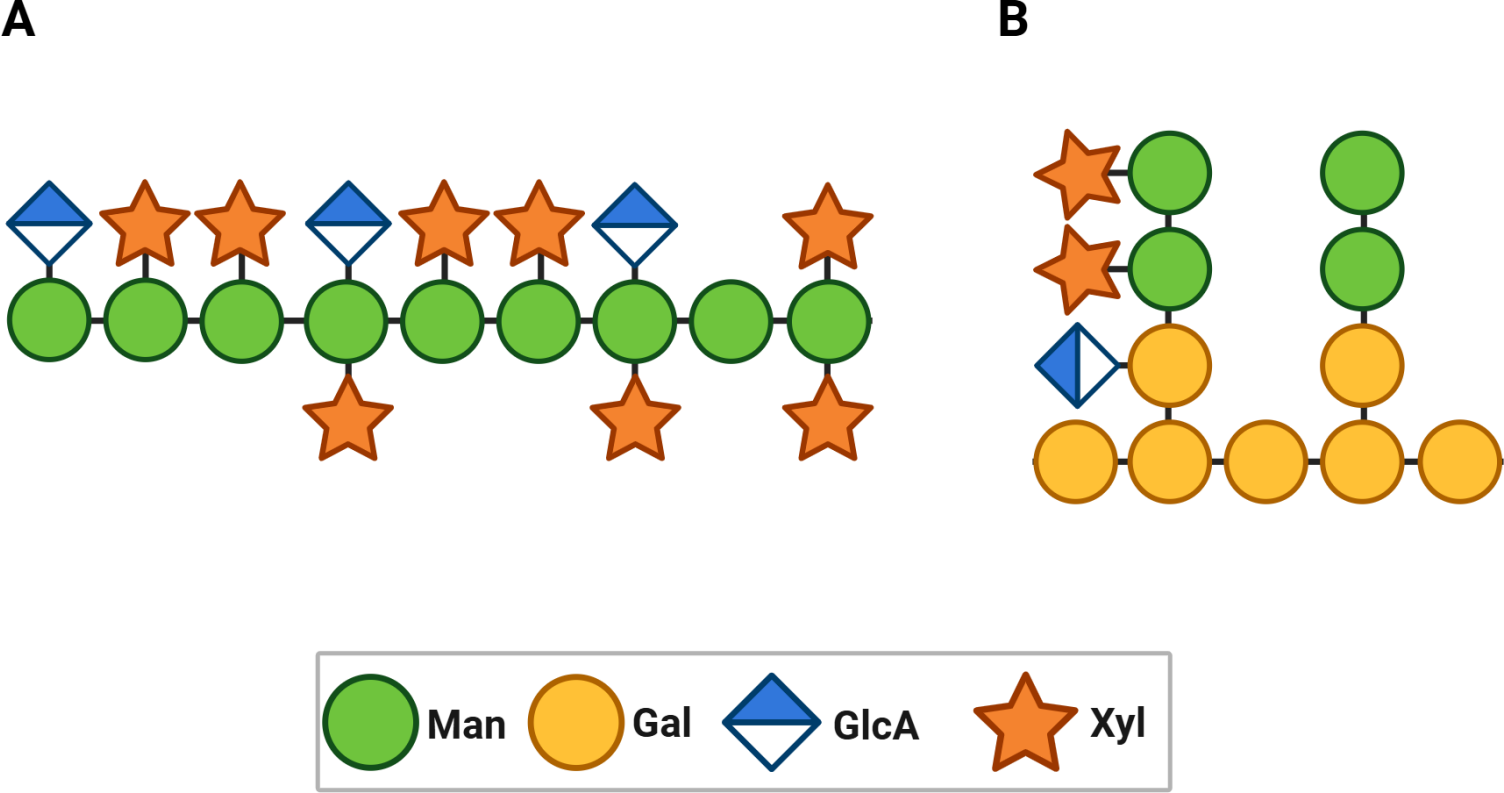
Visualization of a general organization of the polysaccharides constituting the cryptococcal capsule. (**A**) GXM is a branched, heterogeneous polysaccharide mainly composed of an α-(1→3)-mannose backbone with different side chains, including β-D-xylose and β-D-glucuronic acid residues. (**B**) GXMGal is a branched polysaccharide featuring an α-(1→6)-galactan backbone, substituted with galactose, mannose, and xylose residues. Its intricate architecture is implicated in immunomodulatory functions and capsule integrity.

The capsule makes the largest contribution to the cryptococcal virulence composite and is composed of glucuronoxylomannan (GXM) (Fig. 1A) and glucuronoxylomannogalactan (GXMGal) (previously known as galactoxylomannan or GalXM) (Fig. 1B) (10–13). This capsule plays a key role in immune evasion by preventing phagocytosis, interfering with antigen presentation, and impairing T-cell responses (14–16). Other cryptococcal virulence factors include melanin production, the formation of titan cells, the secretion of phospholipases and urease, and the ability to replicate at mammalian body temperatures (17, 18). However, the capsule remains the most distinctive and immunologically relevant element of cryptococcal virulence because it can be targeted directly in vaccine design, given the involvement of polysaccharides in tissue, and in interfering with the immune response. Hence, opsonic antibodies that neutralize the anti-phagocytic and deleterious immunomodulating properties of the cryptococcal capsule could neuter this pathogenic fungus, as evident by the fact that acapsular strains are avirulent (19).

## THE RATIONALE FOR A CONJUGATE VACCINE APPROACH

Because polysaccharides are typically T-cell-independent antigens that elicit poor immunological memory, early vaccine efforts struggled to induce long-term protection using these antigens (20, 21). In this regard, cryptococcal polysaccharide is no exception; polysaccharide-only vaccines are not feasible due to their lack of immunogenicity. In contrast, conjugate vaccines, which are constructed by linking polysaccharide antigens to an immunogenic protein carrier, help overcome this limitation by engaging T-helper cells, promoting class switching, and inducing memory B cell responses (15, 16). This approach has been highly successful in combating encapsulated bacterial pathogens, and there are licensed conjugate vaccines for the prevention of infection with *Streptococcus pneumoniae, Haemophilus influenzae* type B*, and Neisseria meningitidis* (16, 17). Apart from the safety and efficacy, conjugate vaccines have the advantage that specific antibodies to the polysaccharide antigen can be readily measured as a correlate of immunity. Various strategies for developing conjugate vaccines have been applied to cryptococcal vaccine development, with increasing promise (Table 1).

**TABLE 1:** A summary of five conjugate vaccine formulations targeting *Cryptococcus* spp.^*a*^

Vaccine type	Protein component	Polysaccharide component	Strength	Weaknesses	References
Crude polysaccharide filtrate (CnF)	Cryptococcal proteins and cryptococcal mannoproteins	Glucuronoxylomannan and glucuronoxylomannogalactan	Easy and affordable production	Poorly defined composition	(22–26)
Extracellular polysaccharide conjugates	Commercial carrier protein	Glucuronoxylomannan and glucuronoxylomannogalactan	Induce high-titer, functional antibodies	Protective efficacy depends on EPS heterogeneity	(27–29)
Glucuronoxylomannan conjugates	Commercial carrier protein	Glucuronoxylomannan	Target a major capsular virulence factor, induce strong IgG responses	Highly dependent on GXM structural variability	(30–32)
Galactoxylomannan conjugates	Commercial carrier protein	Glucuronoxylomannogalactan	Provide a distinct immunological target	Limited data on polysaccharide structure	(11, 33–35)
Semisynthetic polysaccharide	Commercial carrier protein	Synthetic glucuronoxylomannan decasaccharides	Highly reproducible and well-defined product	Very low yield and very high cost of production	(36, 37)

^*a*^ Each vaccine includes a cryptococcal polysaccharide antigen covalently linked to a protein carrier. Protein components include traditional carriers like tetanus toxoid (TT), diphtheria toxoid (DT), CRM197, or recombinant cryptococcal proteins.

## EARLY FOUNDATIONS: THE CRUDE POLYSACCHARIDE FILTRATE

The first attempt to create a conjugate vaccine for *C. neoformans* failed (22, 23). It is unclear why that early conjugate vaccine failed to elicit protection despite eliciting very high antibody responses, but it is possible that the abundant antibody created a prozone that abolished protective efficacy (24). In the 1990s, the laboratory adopted a different approach, using CneF, a crude culture filtrate rich in shed capsular polysaccharides, as a vaccine. Although compositionally undefined, vaccination with CneF achieved partial protection in mouse models, marked by reduced fungal burden and delayed mortality following challenges with virulent strains (25, 26). The interpretation was that this protection came from proteins in the filtrate eliciting a cell-mediated response as CneF elicited little or no antibody, consistent with its composition. These studies encouraged the development of polysaccharide-containing vaccines and provided the conceptual foundation for more refined conjugate strategies (22, 25, 26).

## GXM CONJUGATES

Given that GXM constitutes over 90% of the cryptococcal capsule, it has naturally become a focal point in vaccine design. Several studies explored purified GXM conjugated to diphtheria toxoid or tetanus toxoid (30, 31). These formulations elicited strong IgG1 and IgG2a responses, resulting in significantly improved survival in murine models, with protection rates ranging from 50% to 80% depending on the challenge strain. GXM-based conjugates also enhanced fungal clearance from the lungs and brain, although the extent of protection was affected by structural variations in the GXM antigen (31, 32). Differences in acetylation, branching patterns, and molecular mass altered immunogenicity and antibody affinity, underscoring the need for either multivalent designs or the identification of conserved antigens for universal protection.

## EXTRACELLULAR POLYSACCHARIDE (EPS) CONJUGATES

Subsequent strategies focused on purifying EPS from *C. neoformans* cultures and chemically conjugating them to protein carriers, such as CRM197 (27, 28). These conjugates consistently induced high titers of IgG against GXM, which promoted macrophage phagocytosis and extended survival in murine models. In multiple studies, EPS-based conjugates provided significant protection against a lethal challenge in vaccinated mice, depending on the strain and challenge dose (29). Passive transfer of immune sera confirmed the protective role of elicited antibodies. Another advancement was reported in a 2025 study, where we optimized EPS purification by integrating EPS profiling and filtration (29). The refined vaccine elicited broad antibody responses and offered significant protection in mice infected with virulent strains of *C. neoformans*. This work emphasized the importance of biophysical uniformity in polysaccharide vaccines, demonstrating that efficacy is linked not only to antigen composition but also to the molecular size of the tested antigens.

## GXMGal CONJUGATES

Although GalXM is less abundant than GXM on a mass basis, it may be more abundant on a molar basis because of its much smaller molecular mass (33). It offers a structurally and immunologically distinct target (11, 34). GXMGal is rich in galactose side chains and has been shown to engage different immune receptors than GXM. The bivalent vaccines containing GXMGal- and GXM-based conjugates showed a promising synergistic effect (35). Mice immunized with GXM-GXMGal bivalent conjugates showed enhanced protection compared to those receiving either antigen alone, with some studies reporting survival rates above 80% and reduced fungal dissemination to the brain (35). These findings suggest that GXMGal may broaden and prolong vaccine-induced immunity and that future formulations may benefit from fine-tuning the ratio and presentation of GXM and GXMGal epitopes (35).

## SEMISYNTHETIC POLYSACCHARIDE-BASED VACCINES

A potentially transformative advance in cryptococcal vaccine development was the development of synthetic oligosaccharides that mimic defined structural motifs of GXM and GXMGal. These semisynthetic constructs are designed using carbohydrate chemistry to reproduce immunodominant regions of the capsule and are then conjugated to carrier proteins (36). In theory, these synthetic oligosaccharides offer the possibility to focus the antibody response on the production of protective antibodies. Studies showed that synthetic GXM oligosaccharide conjugates can elicit epitope-specific, weakly opsonic antibodies (36, 37). Importantly, synthetic vaccines reduce batch-to-batch variability and enable production under defined quality control standards; however, they require further optimization to achieve satisfactory levels of protection. The ability to rationally design synthetic glycoconjugates based on structural immunology data enables precise targeting of conserved glycan epitopes. It may support the parallel development of glycan microarrays for serological diagnostics and vaccine evaluation.

## POLYSACCHARIDE CONJUGATE VACCINES FOR CELLULAR IMMUNE RESPONSE INDUCTION

The existing research highlights the critical role of CD4 + T lymphocytes in controlling cryptococcal infections by naturally produced or passively administered antibodies (38–40). A balanced interplay between cellular and humoral immune responses is essential for controlling fungal load as isolated Th1 or Th17 cellular responses can lead to hyperinflammation and tissue damage and a Th2 humoral response is particularly associated with disease dissemination (33, 34). Therefore, a vaccine that can stimulate Th1, Th17, and Th2 immune responses would be the ideal candidate for a safe and effective vaccine for *Cryptococcus* infections, as demonstrated for other fungal infections (41). This immunoregulatory modulation can be achieved using specific adjuvants, as demonstrated for bacterial polysaccharide-conjugated vaccines (35, 36). Several studies have shown that some *Cryptococcus* proteins are immunogenic and can elicit a Th1 and/or Th17 immune response, leading to increased survival and reduced fungal load. One interesting approach that was never analyzed and should be considered is the polysaccharide conjugation of one of those cryptococcus antigenic proteins to GXM (42–44). This conjugation may be able to induce and equilibrate an immunoreactive response (Th1, Th17, and Th2), leading to fungal elimination without causing hyperinflammation.

## CONCLUSION

Despite the formidable immunological challenges posed by *Cryptococcus* species, recent progress in conjugate vaccine development has yielded promising results (29, 37, 45). From early experiments with crude culture filtrates to recent work with chemically defined EPS conjugates and precision-designed synthetic oligosaccharides, each generation of vaccine has brought the field closer to a clinically viable product (25, 26). The development of a safe and effective human vaccine against *Cryptococcus neoformans* and *Cryptococcus gattii* remains a high-priority goal. The success of some polysaccharide conjugate vaccines makes this approach promising, but further progress will require greater integration of structural glycobiology in vaccine design. Only through collective action can we fully realize the potential of conjugate vaccine strategies and transform the prevention of cryptococcal disease globally (7).
